# Patients’ visual experience during phacoemulsification cataract surgery and associated fear

**DOI:** 10.1186/1756-0500-7-663

**Published:** 2014-09-20

**Authors:** Tanveer Anjum Chaudhry, Amash Aqil, Kanza Aziz, Ammar Asrar Javed, Mohammad Zain Tauqir, Khabir Ahmad

**Affiliations:** Section of Ophthalmology, Department of Surgery, Aga Khan University, Stadium Road, P.O. Box 3500, Karachi, 74800 Pakistan

## Abstract

**Background:**

Data from several published studies indicate that patients undergoing phacoemulsification cataract surgery can experience a variety of visual sensations which can result in fear. This phenomenon has not been studied in Pakistan to-date. We examined the visual experience and its associated fear among patients undergoing phacoemulsification cataract surgery under topical anaesthesia.

**Methods:**

This cross-sectional study was carried out in Aga Khan University Hospital, a tertiary care hospital, in Karachi, Pakistan from August 2010 to July 2011. Adults >18 years of age scheduled to undergo cataract surgery (phacoemulsification with intraocular lens implantation) under topical anaesthesia by a single surgeon were included. A structured questionnaire was used to collect data on socio-demographics, intraoperative visual experiences and subsequent reaction to these sensations. Participants were asked if they experienced visual sensations such as colours, shapes and movements during surgery. Moreover, they were asked if they developed fear due to these sensations.

**Results:**

Fifty three patients (mean age: 60.4 ± 12.4 years) were enrolled. Thirty (56.6%) of them were men and 23 (43.4%) were women. All of them reported having experienced visual sensations during surgery, the most common being light perception (100%), different colours (77.4%), movements of instruments or surgeon’s hands (37.7%) and different shapes (7.5%) such as circles, clouds and patches. The most common colours perceived included white (46.2%), blue (35.8%), red (30.2%) and yellow (30.2%). One out of every four (26.4%) participants reported having developed fear due to these visual sensations. Only 4 (7.5%) reported having received preoperative counselling regarding such sensations.

**Conclusion:**

Patients in our study experienced a variety of visual sensations during cataract surgery under topical anaesthesia. The prevalence of frightening visual sensations is higher than that reported in all previous published studies on the subject and needs to be addressed through targeted interventions.

## Background

Cataract is the leading cause of avoidable blindness and visual impairment in the world [1]. As a result, cataract surgery is one of the most common surgical procedures performed worldwide [2]. The two most common surgical procedures for removing cataracts are phacoemulsification and extra-capsular cataract extraction (ECCE). Most of the cataract surgeries can be performed using either local or topical anesthesia. Only a small proportion of these patients require general anesthesia. The choice between topical and local anesthesia generally varies according to the preference and expertise of the surgeon and patients’ characteristics such as age, personality type and pain perception [3].

A series of studies have reported that a significant proportion of patients undergoing cataract surgery may experience a multitude of visual sensations intraoperatively [4–11]. These sensations may be affected by the type of anesthesia as patients who receive topical anesthesia (TA) are able to see more in comparison with patients who are administered regional anesthesia (RA). This is mainly due to the fact that TA does not usually affect visual function whereas RA, such as a retrobulbar injection, has been shown to temporarily compromise the function of the optic nerve [9].

The study of subjective visual experiences of patients during phacoemulsification cataract surgery has attracted considerable attention over the last two decades. However, this phenomenon has not yet been explored in a developing country’s setting. We conducted a cross-sectional study to examine the visual experience and its associated fear among patients undergoing phacoemulsification cataract surgery under topical anesthesia.

## Methods

A cross-sectional study was conducted from August 2010 to July 2011 at the Aga Khan University Hospital (AKUH), Karachi. The inclusion criteria was adults >18 years of age scheduled to undergo cataract surgery (phacoemulsification with intraocular lens implantation) under topical anaesthesia by a single surgeon. Patients with communication barriers were excluded. The topical anaesthetic agents used were proparacaine hydrochloride 2% eye drops/gel or lignocaine 2% eye drops. The operating microscope used was Carl Zeiss S88. None of these patients received sedation in the pre-, intra- and post-operative periods, as is the routine practice in our unit.

Ethics approval was obtained from the Ethical Review Committee of the Aga Khan University, Karachi. Informed consent was obtained from all participants. Each participant was interviewed 10–20 minutes after completion of the cataract surgery. A structured questionnaire was used to collect data, which included questions about the characteristics of the subjects, their intraoperative visual experience and subsequent reaction to these sensations. Self-perceived visual sensations were examined by asking the question: which of the following did you perceive in the operated eye during surgery? Light, colours, shapes, movements of instruments, movements of surgeon’s hands or none of these. Those who reported colours and movements were asked to specify the colour, or type of movement, respectively. Moreover, subjects were asked if they experienced any fear due to the different visual stimuli experienced during the procedure. Data were entered and analysed using SPSS version 19.0. Descriptive analysis was done to generate frequencies and proportions. Chi-square test or Fisher's exact test was used, as appropriate, for significance testing. A p-value of <0.05 was considered statistically significant.

## Results

A total of 53 patients undergoing cataract surgery under topical anaesthesia were interviewed. The mean (±SD) age of study participants was 60.4 ± 12.4 years. Thirty (56.6%) of them were men and 23 (43.4%) were women (Table 1). All of them reported having experienced visual sensations during surgery (Table 2). The most common sensations experienced were light perception (100%), different colours (77.4%), movements of instruments or surgeon’s hands (37.7%) and different shapes (7.5%) such as circles, clouds and patches. Of the different colours perceived, the most common ones were white (46.2%), blue (35.8%), red (30.2%) and yellow (30.2%). The overall prevalence of fear associated with visual sensations was 26.4%. There was no significant association between the presence of fear and covariates such as age, sex, level of education, previous eye surgery or visual acuity (Table 3). Only 4 (7.5%) participants reported having received preoperative counselling regarding possible visual sensations during surgery and all of them thought it was helpful in reducing their fear.

**Table 1:** **Socio-demographic characteristics of study participants (n = 53)**

Variable		Count	%
**Age group**	< 60 years	25	47.2
	≥ 60 years	28	52.8
**Sex**	Male	30	56.6
	Female	23	43.4
**Level of education**	Below primary	7	13.2
	Primary	2	3.8
	Secondary	13	24.5
	Graduate	24	45.3
	Post graduate	7	13.2
**Mother tongue**	Balochi	3	5.7
	Pakhtoon	3	5.7
	Punjabi	3	5.7
	Sindhi	10	18.9
	Urdu	25	47.2
	Other	9	17.0
**Preoperative visual acuity**	≥ 6/18	37	69.8
	< 6/18	16	30.2

**Table 2:** **Visual sensations experienced by patients (n = 53) during cataract surgery under topical aesthesia**

***Type of visual experience***	Frequency	%
**Able to see anything during surgery**	**53**	**100.0**
**Able to see light**	**53**	**100.0**
**Colours seen**	**41**	**77.4**
White	18	46.2
Blue	19	35.8
Red	16	30.2
Yellow	16	30.2
Green	13	24.5
Orange	8	15.1
Pink	2	3.8
**Movements**	**20**	**37.7**
Water	10	18.9
Instruments	10	18.9
Fingers\hands	9	17.0
**Different shapes**	**4**	**7.5**

**Table 3:** **Prevalence of frightening visual sensations among study participants**

Variable		Interviewed (n = 53)	Individuals with frightening visual sensations		***P***
**All**		**53**	**14**	**26.4**	
**Age group**	< 60 years	25	8	32.0	0.384
	≥ 60 years	28	6	21.4	
**Sex**	Male	30	7	23.3	0.561
	Female	23	7	30.4	
**Level of education**	Secondary or less	22	8	36.4	0.166
	More than secondary	31	6	19.4	
**Previous history of eye surgery**	Yes	38	12	31.6	0.175
	No	15	2	13.3	
**Preoperative visual acuity**	≥ 6/18	37	12	32.4	0.131
	<6/18	16	2	12.5	
**Type of visual sensation**	Light*	53	14	26.4	…
	Colours	41	12	29.3	0.480
	Movements	20	7	35.0	0.341
	Different shapes	4	1	25.0	1.00

*All patients reported seeing light during surgery.

## Discussion

This is one of the first studies to examine visual sensations during cataract surgery in a developing country’s setting. Consistent with previous literature, our study indicates that patients experience a wide range of visual sensations during cataract surgery under topical anaesthesia which included light, colours, movements and different shapes [4–11].

In our study, one out of every four subjects reported having experienced fear due to the sensations they experienced. This prevalence of frightening visual sensations (26.4%) is higher than that reported in all previous published studies on this subject [6, 7, 11–13]. A possible reason for higher than expected prevalence of fear in our study population could be the fact that TA was used in all cases. Another reason could be the lack of pre-operative counselling in our setting. Previous literature has reported that fear is a response to perceived threat which can be minimised by appropriate health education interventions [14]. In one such study, Voon and colleagues reported a mean fear score of 0.3 in patients who received preoperative counselling compared to a mean fear score of 0.9 in those who did not [13]. It is well-known that topical anaesthesia preserves optic nerve function [15] and thus patients can experience a vast range of visual stimuli. Therefore, anaesthesia-specific counselling could be of value for every patient scheduled to undergo phacoemulsification cataract surgery, in particular, regarding various possible stimuli such as touch of water, noise, movements etc. Information on frightening visual sensations could be included in cataract surgery information leaflets.

An interesting finding of our study was that there was no statistically significant association between fear and covariates such as age, gender, level of education, previous eye surgery and pre-operative visual acuity. This lack of association may be explained, at least in part, by the relatively small number of cases selected through convenience sampling. Some variability in visual sensations would be expected in a more representative sample and future studies should address this issue in an effective way and should include a control population (undergoing cataract surgery under regional anaesthesia) as well.

## Conclusion

Our study shows that patients undergoing cataract surgery under topical anaesthesia experience a wider range of visual stimuli. The prevalence of frightening visual sensations is higher than that reported in all previous published studies on the subject and fear reduction interventions are needed.

## References

[1] Pascolini D, Mariotti SP (2012). Global estimates of visual impairment: 2010. Br J Ophthalmol.

[2] Taylor H (2000). Cataract: how much surgery do we have to do?. Br J Ophthalmol.

[3] Tauqir MZ, Chaudhry TA, Mumtaz S, Ahmad K (2012). Knowledge of patients’ visual experience during cataract surgery: a survey of eye doctors in Karachi, Pakistan. BMC Ophthalmol.

[4] Murdoch IE, Sze P (1994). Visual experience during cataract surgery. Eye (Lond).

[5] Rengaraj V, Radhakrishnan M, Au Eong KG, Saw SM, Srinivasan A, Mathew J, Ramasamy K, Prajna NV (2004). Visual experience during phacoemulsification under topical versus retrobulbar anesthesia: results of a prospective, randomized, controlled trial. Am J Ophthalmol.

[6] Au Eong KG, Low CH, Heng WJ, Aung T, Lim TH, Ho SH, Yong VS (2000). Subjective visual experience during phacoemulsification and intraocular lens implantation under topical anesthesia. Ophthalmology.

[7] Au Eong KG, Lim TH, Lee HM, Yong VS (2000). Subjective visual experience during phacoemulsification and intraocular lens implantation using retrobulbar anesthesia. J Cataract Refract Surg.

[8] Au Eong KG (2002). 6th Yahya Cohen Lecture: visual experience during cataract surgery. Ann Acad Med Singapore.

[9] Yaylali SA, Akcakaya AA, Erbil HH, Candemir B, Mesci C, Acar H (2012). The relationship between optical coherence tomography patterns, angiographic parameters and visual acuity in age-related macular degeneration. Int Ophthalmol.

[10] Tan CS, Au Eong KG, Kumar CM (2005). Visual experiences during cataract surgery: what anaesthesia providers should know. Eur J Anaesthesiol.

[11] Prasad N, Kumar CM, Patil BB, Dowd TC (2003). Subjective visual experience during phacoemulsification cataract surgery under sub-Tenon’s block. Eye (Lond).

[12] Haripriya A, Tan CS, Venkatesh R, Aravind S, Dev A, Au Eong KG (2011). Effect of preoperative counseling on fear from visual sensations during phacoemulsification under topical anesthesia. J Cataract Refract Surg.

[13] Voon LW, Au Eong KG, Saw SM, Verma D, Laude A (2005). Effect of preoperative counseling on patient fear from the visual experience during phacoemulsification under topical anesthesia: multicenter randomized clinical trial. J Cataract Refract Surg.

[14] Grillon C (2008). Models and mechanisms of anxiety: evidence from startle studies. Psychopharmacology.

[15] Tranos PG, Wickremasinghe SS, Sinclair N, Foster PJ, Asaria R, Harris ML, Little BC (2003). Visual perception during phacoemulsification cataract surgery under topical and regional anaesthesia. Acta Ophthalmol Scand.

